# Modulating Carrier Type for Enhanced Thermoelectric Performance of Single-Walled Carbon Nanotubes/Polyethyleneimine Composites

**DOI:** 10.3390/polym11081295

**Published:** 2019-08-02

**Authors:** Xiao-Xi Peng, Xuan Qiao, Shuai Luo, Jun-An Yao, Yun-Fei Zhang, Fei-Peng Du

**Affiliations:** School of Materials Science and Engineering, Wuhan Institute of Technology, Wuhan 430205, China

**Keywords:** single-walled carbon nanotubes, polyethyleneimine, thermoelectric properties, carrier type

## Abstract

Thermoelectric (TE) generators consisting of flexible and lightweight *p*- and *n*-type single-walled carbon nanotube (SWCNT)-based composites have potential applications in powering wearable electronics using the temperature difference between the human body and the environment. Tuning the TE properties of SWCNTs, particularly *p*- versus *n*-type control, is currently of significant interest. Herein, the TE properties of SWCNT-based flexible films consisting of SWCNTs doped with polyethyleneimine (PEI) were evaluated. The carrier type of the SWCNT/PEI composites was modulated by regulating the proportion of SWCNTs and PEI using simple mixing techniques. The as-prepared SWCNT/PEI composite films were switched from *p*- to *n*-type by the addition of a high amount of PEI (>13.0 wt.%). Moreover, interconnected SWCNTs networks were formed due to the excellent SWNT dispersion and film formation. These parameters were improved by the addition of PEI and Nafion, which facilitated effective carrier transport. A TE generator with three thermocouples of *p*- and *n*-type SWCNT/PEI flexible composite films delivered an open circuit voltage of 17 mV and a maximum output power of 224 nW at the temperature gradient of 50 K. These promising results showed that the flexible SWCNT/PEI composites have potential applications in wearable and autonomous devices.

## 1. Introduction

Thermoelectric (TE) energy-harvesting generators, composed of multiple *p*- and *n*-type TE materials that are electrically and thermally connected in series and in parallel, respectively, can directly convert heat energy into electric energy and vice versa under a temperature gradient without moving parts. These generators are also quiet, exhibit long lifetimes, and are environmentally friendly which gives them wide applicability in power generation for frontier electronic devices [1,2,3]. The performance of TE materials is evaluated by a dimensionless figure of merit, *ZT*, through the equation *ZT* = *S^2^σT*⁄*κ* (where *S* is the Seebeck coefficient, *σ* is the electrical conductivity, *κ* is the thermal conductivity, and *T* is the absolute temperature), wherein *S*^2^*σ* is defined as power factor (PF) [4,5,6]. The Seebeck coefficient can be positive (holes, *p*-type) or negative (electron, *n*-type) depending on the main charge carrier type [7,8,9]. Inorganic semiconductors, such as Bi_2_Te_3_, PbTe, and Sb_2_Te_3_, are often used as TE materials due to their high *ZT* values. However, their relatively high cost, high toxicity, and poor processability impede their application in flexible electronics [10,11]. Therefore, it is necessary to develop advanced *p*- and *n*-type TE materials for flexible electronic devices.

Recently, carbon nanotubes (CNTs) and their composites have been intensively studied and utilized as TE materials for energy harvesting due to their mechanical flexibility, light-weight characteristics, and facile processability [11,12,13,14,15]. By improving the dispersion of CNTs in CNT/polymer composites, the TE performance of the composite could be effectively enhanced. For example, through non-covalent modification of the surface of single-walled carbon nanotubes (SWCNTs) with polymer, He et al. prepared a substrate-free and well-dispersed polythiophene/SWCNT composite, which exhibited a *ZT* of 3.1 × 10^−2^ with a PF of 28.8 μW/(m·K^2^) at room temperature [16]. Kim et al. improved the dispersion state of SWCNTs in composite fibers via simple wet-spinning of SWCNT/poly(vinylidene fluoride) (PVDF) pastes and achieved optimized PFs for the *p*- and *n*-type SWCNT/PVDF composite fibers of about 378 and 289 μW/(m·K^2^), respectively [17]. It was noted that the simultaneous increase in electrical conductivity and the absolute value of the Seebeck coefficient is a useful way to enhance the PF, as observed for CNT-based TE materials, such as polyaniline (PANI)/CNTs [18] and paper-based CNT composites [19]. Yao et al. reported that the carrier mobility, which contributes to the increase in electrical conductivity, of PANI/SWCNT composites increased with increasing SWCNT content, which was ascribed to the ordering of PANI chains induced by π–π interactions between SWCNTs and PANI [18,20]. Most of the CNT-based materials reported in the past few decades have been *p*-type, due to oxygen impurities [21]. Recently, unstable *n*-type CNT composites have attracted great attention due to their role in fabricating complete TE generators [22,23,24]. Doping CNTs with *n*-type polymers or molecules containing electron donor groups, such as poly (vinyl pyrrolidone) [25], poly (vinyl chloride), PVDF [26], polyethyleneimine (PEI) [23,27], NaBH_4_ [23], and N_2_H_4_ [28], has been considered a promising strategy for obtaining *n*-type CNT-based TE materials. Yu et al. reported that treatment with the strong reducing agent NaBH_4_ followed by lamination improved the air stability of *n*-type PEI-doped CNT composites, yielding Seebeck coefficients as large as −80 μV/K [23]. PEI is a relatively simple, effective, and low-cost *n*-type doping agent for CNTs. Also, branched PEI can be physically adsorbed onto the sidewalls of CNTs by facile techniques such as solution mixing [22,25], spin coating [28], or layer-by-layer (LbL) assembly [29]. Therefore, PEI-doped CNT composites have been widely used as *n*-type legs for flexible TE generators [22,23,30]. While most studies only report the instability of the composite in the air and methods to improve the air stability of PEI-doped CNTs [21,23,30], little work has been focused on the effect of PEI on the dispersion of SWCNTs and the comprehensive TE performance of SWCNT/PEI composites. Our recent findings demonstrated that a small amount of PEI improves the dispersion and film-forming properties of SWCNTs, causing a simultaneous increase in the electrical conductivity and Seebeck coefficient of *p*-type SWCNT/PEI composites as compared to pure SWCNTs.

In this work, both *p*- and *n*-type SWCNT/PEI composite TEs were developed by modulating the proportion of SWCNTs to PEI using simple mixing techniques. Through controlling the carrier type of SWCNT/PEI composites, the prepared composites exhibited maximum Seebeck coefficients of 49 and −40 μV/K with electrical conductivities of 210 and 170 S/cm for *p*- and *n*-type legs, respectively. Furthermore, a flexible TE generator comprising *p*- and *n*-type SWCNT/PEI composites has been assembled to demonstrate the TE energy conversion ability of these composites.

## 2. Experimental

### 2.1. Materials

SWCNTs were purchased from the Shenzhen Nanotech Port Corporation (Shenzhen, China) at >83% purity, > 15 μm in length, and 1–3 nm in diameter. Branched PEI (molecular weight: 1800, 99%) was purchased from Aladdin Industrial Corporation (Shanghai, China). The Nafion solution (5.2 wt.%) was bought from DuPont Corporation (Wilmington, DE, USA). All other analytical reagents were purchased from the Sinopharm Chemical Reagent Corporation (Shanghai, China). All of the chemicals were used as received without further purification.

### 2.2. Preparation of Flexible p- and n-Type Single-Walled Carbon Nanotube/Polyethyleneimine (SWCNT/PEI) Films

In order to obtain a SWCNT/PEI composite film, 12.0 mg SWCNTs and certain amount of PEI were dispersed in 6.0 mL ethanol to obtain a suspension. The PEI content was varied from 0.0 to 20.0 wt.% to adjust the TE performance. Accordingly, the concentration of PEI was selected as 0.0 wt.%, 2.0 wt.%, 7.0 wt.%, 13.0 wt.%, 15.0 wt.% and 20.0 wt.%, named as SWCNT/PEI-0, SWCNT/PEI-2, SWCNT/PEI-7, SWCNT/PEI-13, SWCNT/PEI-15 and SWCNT/PEI-20, respectively. Then, 0.05 mL of a 1.0 wt.% Nafion solution was added to improve the dispersion of the mixture. The mixture was then stirred at room temperature for 12 h to form a homogeneous, well-distributed solution. Finally, the SWCNT/PEI composite film was prepared via vacuum filtration of the SWCNT-PEI dispersion onto a PVDF membrane (0.22 μm) and subsequent drying at 55 °C overnight in a vacuum oven (Shanghai Soxpec Instrument,, Shanghai, China). The thickness of the obtained SWCNTs/PEI film was approximately 30 μm, which was measured with a thickness gauge (Resolution: 1 μm).

### 2.3. Thermoelectric Device Fabrication

The as-prepared *p*- and *n*-type SWCNT/PEI films were cut into rectangular shapes (5 × 20 mm) and pasted onto a polypropylene (PP) substrate. Then, *p*- and *n*-type SWCNT/PEI films were alternately linked with conductive silver paste to fabricate a flexible TE generator consisting of 3 *p*-*n* junctions. Finally, the TE generator was sealed with a transparent PP film. A temperature gradient was used to measure the output voltage and current of the TE generator. These parameters were also measured under different load resistances.

### 2.4. Characterization

Raman spectra were recorded using a Raman spectrometer (DXR, USA) at a wavelength of 532 nm. The surface microstructures of the SWCNT/PEI composite films were characterized using scanning electron microscopy (SEM, Phillips XL30). Transmission electron microscopy (TEM) images were recorded on a JEOL 2010 electron microscope at an accelerating voltage of 200 kV. X-ray diffraction (XRD) patterns (from 10° to 90° of 2*θ*, Cu-K_α_ radiation, λ = 1.54 Å) were obtained using a New D8-Advance/Bruker-AXS (Karlsruhe, Germany) powder X-ray diffractometer operating at 40 kV and 30 mA, with a scanning rate of 6°/min and a scanning step of 0.02. The electrical conductivity and Seebeck coefficients were measured using a TE parameter test system (Namicro-III, Wuhan Schwab Instruments, Wuhan, China). When measuring the Seebeck coefficient, all of the samples had similar dimensions of 14.0 mm × 14.0 mm × 30 μm. The temperature gradient was set from 0.3 to 3.5 °C along the longitudinal direction of the samples. The PF was obtained via the formula PF = *S^2^σ*. The Hall effect measurement was recorded under a magnetic field of 0.68 *T* using a Hall effect test system (HET-RT, Wuhan Schwab Instruments, Wuhan, China). The voltage and power generated by the TE generator were measured with a home-built system in an ambient environment and calculated by the temperature difference (Δ*T*, provided by Peltier devices and determined by *T*-type thermocouples) and the change in thermal electrical voltage (Δ*V*, measured with Keithley 2000 multimeter). Then, the output power of the TE generator was obtained as a function of the load resistance (*R*) using the equation *P* = *V^2^*/*R* [31].

## 3. Results and Discussion

**Figure 1:**
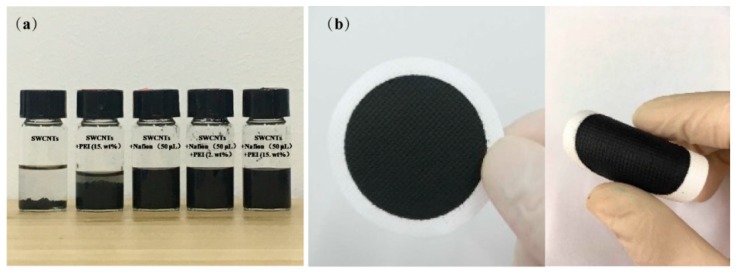
Photograph of SWCNT dispersions containing PEI or Nafion (**a**) and the SWCNT/PEI composite containing 2.0 wt.% PEI (**b**).

Figure 1a indicates that the dispersion ability of SWCNTs was improved by the addition of PEI and Nafion. Also, all of the prepared films were smooth and compacted and showed great flexibility (Figure 1b), wherein they could endure bending without destruction, which is beneficial for the assembly of these films into TE devices.

**Figure 2:**
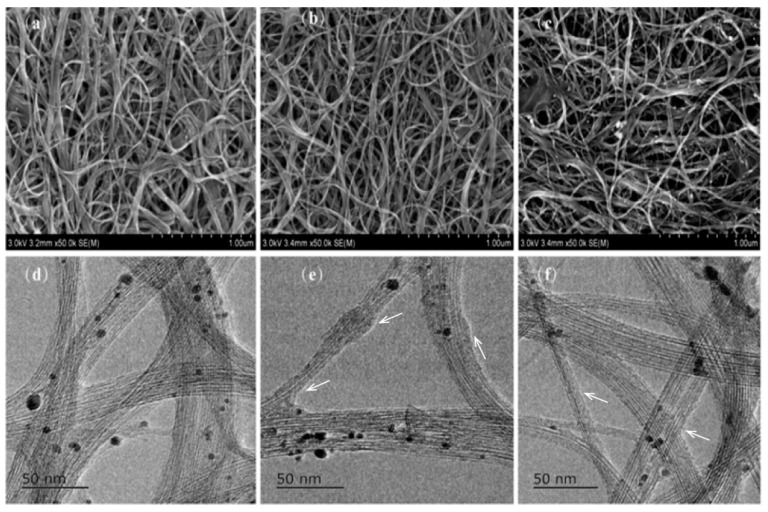
Transmission electron microscope (TEM) and scanning electron microscope (SEM) images of single-walled carbon nanotubes (SWCNTs) doped with different amount of polyethyleneimine (PEI). (**a**,**d**) 0 wt.%, (**b**,**e**) 2 wt.%, (**c**,**f**) 15 wt.%.

Figure 2a–c displays typical SEM images of the SWCNT/PEI composites containing 0, 2, and 15 wt.% PEI, respectively. In these images, it is clearly seen that numerous interconnected tubes form a porous network due to the excellent flexibility of the individual SWCNTs. The TEM images of the composites (Figure 2e–f) show that the SWCNTs’ surface was mainly coated with PEI while some parts were naked (indicated by arrows), implying strong binding between the SWCNTs and PEI. The black patches visible in the TEM images might be ascribed to Nafion adsorption on the surface of the tubes.

**Figure 3:**
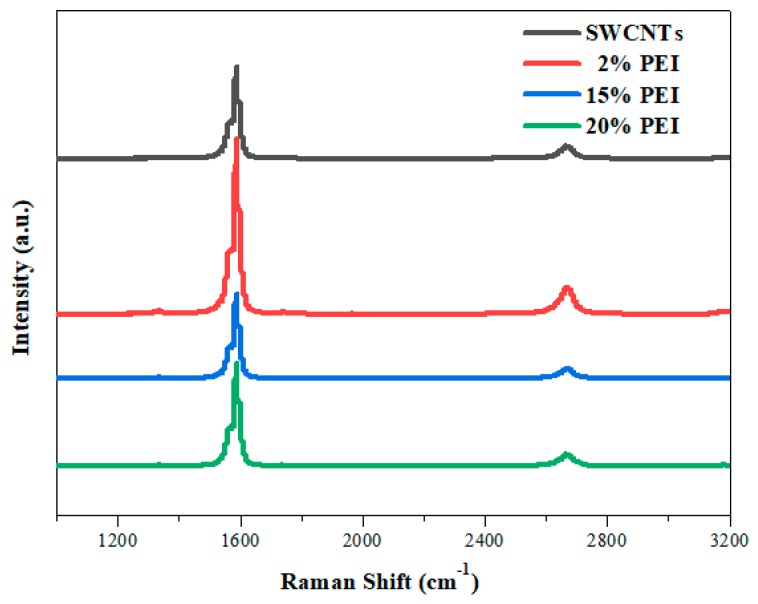
Raman spectra of pristine SWCNTs and PEI-doped SWCNTs.

Raman spectrometry was used to examine the defects and disordered structures of the carbo*n*-based materials. Raman spectra of the pristine SWCNTs and the PEI-doped SWCNTs are shown in Figure 3. The G band (in-plane stretching, E_2g_ mode) can be attributed to the vibration of sp^2^ bonded carbon atoms while the D band corresponds to the vibration of carbon atoms with dangling bonds, which reflects the defects and the disordered structures of the SWCNTs [23,31,32,33]. The D bands of both the pristine and PEI-doped SWCNTs were found at approximately 1337 and 2670 cm^−1^ (D_1_ and D_2_, respectively), while the G bands were located at about 1587 cm^−1^. Usually, the intensity ratio of D:G (I_D_:I_G_) qualitatively indicates the amount of PEI that is attached to the carbon nanotubes. In this work, as the PEI content increased from 0 to 20 wt.%, the intensity ratio was similar and around 0.015. These values are quite small compared with the reported results [23,34]. The SWCNTs employed here were not functionalized for the purpose of high electrical conductivity; therefore, we believe that the interaction between PEI and the SWCNTs was non-covalent.

**Figure 4:**
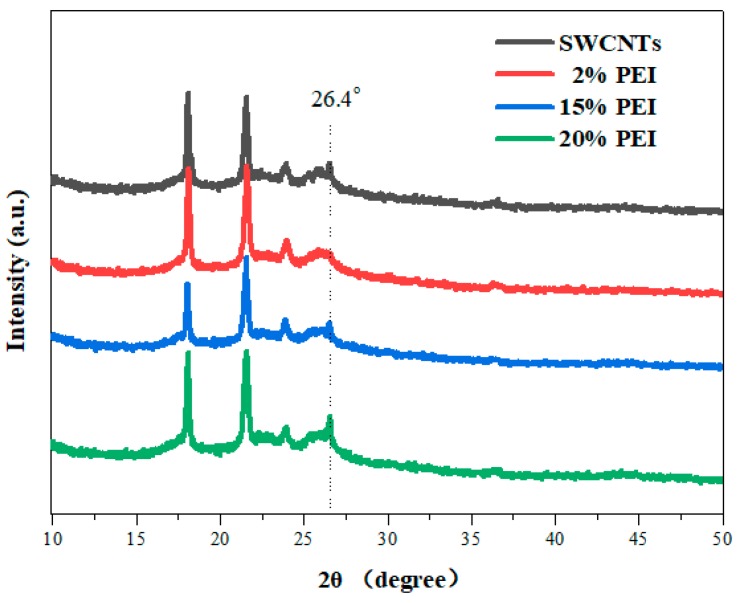
X-ray diffraction (XRD) patterns of pristine SWCNTs and SWCNT/PEI composites.

XRD measurements were employed to identify the crystalline phase and structure of pristine SWCNTs and SWCNT/PEI composites (Figure 4). The XRD patterns of the pure SWCNTs exhibit a typical diffraction peak at 2*θ* = 26.5°, which corresponds to graphite reflection [18,20]. The diffraction peak at 2*θ* = 26.4° of the SWCNTs was observed in all four samples, indicating that their crystalline structure remains intact and was not destroyed during the composite preparation process. A SWCNTs bundle diffraction peak below 16° was not found, which might be ascribed to low quality of SWCNTs. The peaks located at 18.1, 21.6, 23.9, and 36.8° (2*θ*) were assigned to Nafion [35,36] and these peaks were observed in all four samples. Compared with pristine Nafion, the crystallinity of Nafion mixed with SWCNTs was increased, suggesting that strong interfacial interactions between SWCNTs and Nafion induced the crystallization of Nafion [29]. The Nafion present in the composites might act as a barrier to permeating oxygen, imparting long-term air stability to the composites.

**Figure 5:**
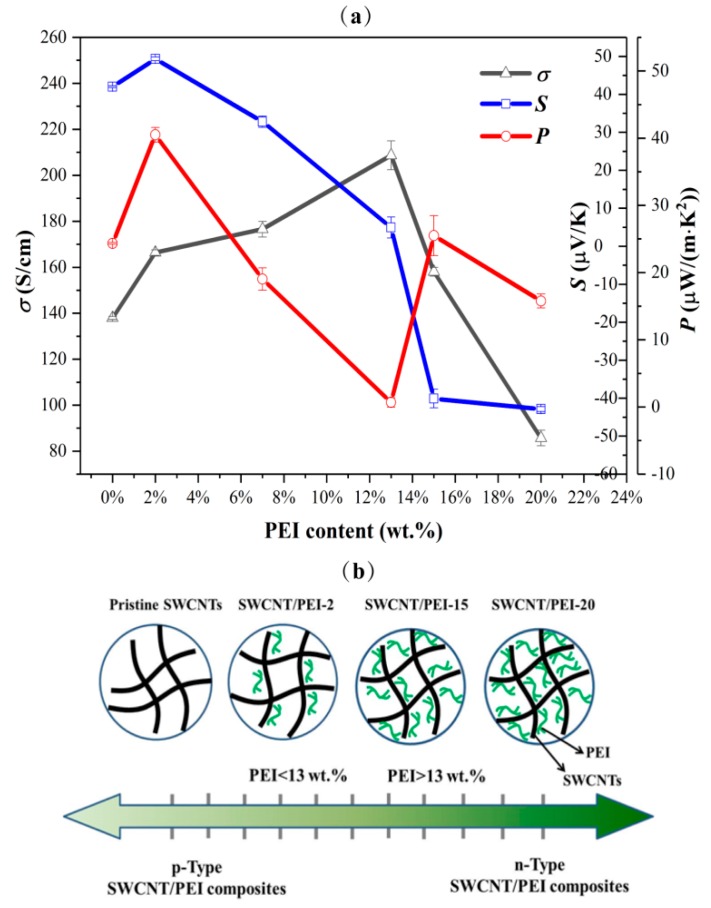
(**a**) The influence of PEI content on the thermoelectric properties of SWCNT/PEI composites. (**b**) Schematic representation of the carrier type of SWCNT/PEI composites tuned by PEI content.

Figure 5a shows the influence of PEI content on the electrical conductivity, Seebeck coefficient, and PF of SWCNT/PEI composite films. With an increase in PEI content, the electrical conductivity of the composite first shows an increasing trend, reaching maximum conductivity at 13 wt.% PEI content, followed by a rapid decrease. The increase in electrical conductivity might be attributed to the formation of an interconnected SWCNT network, due to improved dispersion and film formation prompted by the addition of PEI and Nafion, which facilitates effective carrier transport. Since PEI is an electrical insulator, when a large amount of PEI is coated on the surface of the SWCNTs, it may prevent carrier transport across the junctions between the SWCNTs, resulting in a reduction in electrical conductivity [37]. Amine-rich PEI was adsorbed onto the surface of the SWCNTs where it acts as an electron donor, which changed the carrier type of the SWCNT/PEI composites from *p*- to *n*-type when high amounts of PEI were used (Figure 5b) [22,38]. The composite films exhibited *p*-type TE behavior when the PEI content was below 13 wt.%, and *n*-type behavior when the PEI content was above 13 wt.%, as shown in Figure 5a, where the maximum Seebeck coefficients of the *p*- and *n*-type composites are 43 μV/K (SWCNT/PEI-2) and −40 μV/K (SWCNT/PEI-15), respectively. Consequently, the maximum PFs of the *p*- and *n*-type composites reached 40.5 and 25.5 μW/(m·K^2^), respectively.

**Figure 6:**
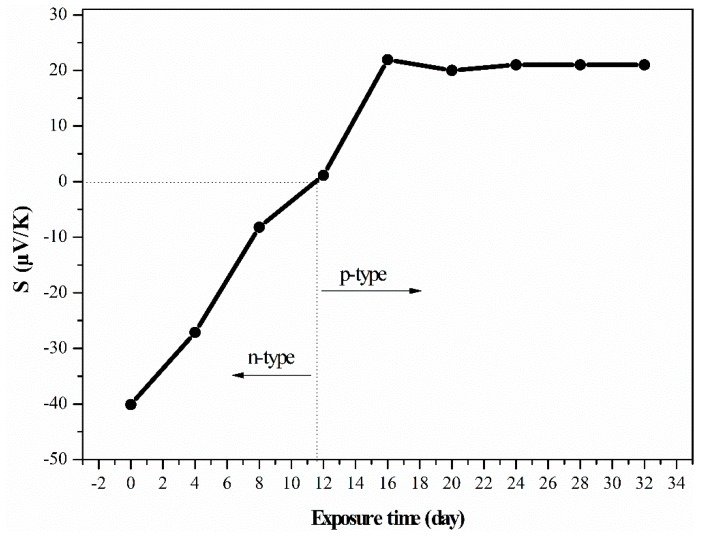
Seebeck coefficient of the SWCNT/PEI-15 composite as a function of exposure time in an ambient environment.

Air stability is a critical issue for *n*-type PEI-doped CNT TE materials. In previous works, negative Seebeck coefficient values of PEI-doped CNTs became positive after 10 h [21] or 9 days [22]. Song et al. [22] and Yu et al. [23] reported that an oxygen adsorption and oxidation reaction may be taking place in the *n*-type PEI-doped CNTs, which changes the sample from n to *p*-type. In our work, the prepared *n*-type SWCNTs/PEI sample is also suffering the problem of being oxidized to *p*-type. For example, the Seebeck coefficient of SWCNT/PEI-15 as a function of exposure time in an ambient environment has been examined and the results are shown in Figure 6. It can be observed that the Seebeck coefficient value of SWCNT/PEI-15 decreases dramatically after it is exposed to air for the given period of time. The Seebeck coefficient value began to change from negative to positive (1.3 μV/K) after 12 days of exposure and became stable (21.9 μV/K) after 16 days of exposure. Therefore, for the practical application of PEI-doped CNTs, strategies such as providing a protective coating, sealing or lamination should be considered to preserve the *n*-type characteristics of the composites [22,23].

The Hall effect measurement was used to study the carrier concentration and carrier mobility of SWCNT/PEI samples (see Table 1). Compared with pristine SWCNTs, the carrier concentration of SWCNT/PEI composites increased with the addition of PEI. This is most likely due to the action of the amine-rich PEI, which acts as an electron donor, wherein a large amount of lone pair electrons might be donated to the PEI-doped SWCNTs. However, the carrier mobility of SWCNT/PEI composites decreased with the addition of PEI compared with pristine SWCNTs. This could be due to the high density of amine groups with lone pairs of electrons acting as scattering points for the carrier [25,38,39]. Therefore, the electrical conductivity of heavily doped SWCNT/PEI composites was reduced because of the low carrier mobility.

**Figure 7:**
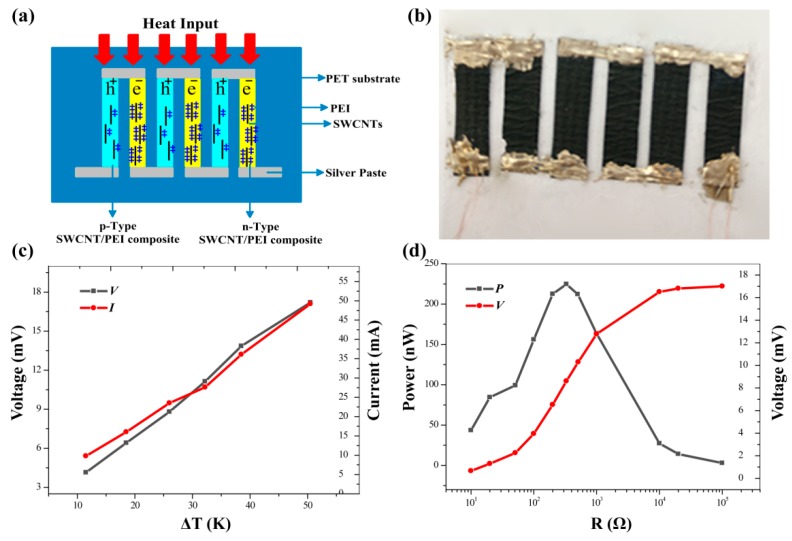
(**a**) Scheme and (**b**) demonstration of the TE generator comprised of three thermocouples. (**c**) Output voltage generated by a three *p*-*n* junction couples as a function of temperature gradient. (**d**) Output voltage and power as functions of different load resistances for the prepared device with a temperature gradient of 50 K.

A flexible TE generator was assembled by electrically connecting the *p*-type SWCNT/PEI-2 film with the *n*-type SWCNT/PEI-15 film using silver paste. Figure 7a,b depict the schematic representation and demonstration of the prepared flexible TE generator consisting of three *p*-*n* junctions pasted on a PP substrate, respectively. The temperature gradient (50 K) provided an output voltage of ~6 mV for one *p*-*n* junction couple in our experiment. Figure 7c shows the relationship between the total output voltage and current versus temperature for the TE generator with three *p*-*n* junctions. As the temperature gradient increased, both output voltage and current increased linearly, which is ascribed to the Seebeck effect. When temperature gradients of 26 and 50 K were separately applied to the prepared TE generator, the voltage was approximately 8.8 and 17 mV, respectively. Power measurements were conducted for several different load resistances and the results are shown in Figure 7d. It was seen that the power reached maximum output (224 nW) when the load resistance was 330 Ω, which is close to the resistance of the TE generator. Despite the fact that the prepared TE generator was composed of only three *p*-*n* junctions, the power output is higher than that of generators composed of six *p*-*n* junctions or *p*- and *n*-type SWCNTs [22,38], which can be attributed to the high PF of both the *p*- and *n*-type SWCNT/PEI composites that were synthesized and investigated in this work.

The output voltage (8.6 mV) is lower than that of the open circuit voltage (17 mV) of the generator at the load resistance of 330 Ω and the temperature gradient of 50 K. This is mainly due to the relatively high internal resistance of the TE generator [22]. Although the output voltage here is low, it can be scaled up by increasing the number of *p*-*n* junctions, making the fabrication of flexible TE generators with much higher power outputs feasible.

Table 2 shows a comparison of the thermoelectric performance obtained from this work as compared with the results of some typical flexible thermoelectric devices. It is found that the output voltage and power of SWCNTs/PEI devices with small dimensions prepared in this work are superior to most of the reported low toxic flexible composite devices, which exhibit high potential for producing flexible thermoelectric devices.

In general, regulating the proportion of SWCNTs and PEI can modulate the carrier type and balance the thermal conductivity and electrical conductivity of the SWCNTs/PEI composites. The SWCNTs/ PEI composites and the preparation method demonstrated in this study are environmentally friendly, cost effective, and easy for large scale production. The prepared SWCNTs/PEI composites exhibited high Seebeck coefficient and relative high power factor, demonstrating a great potential in the preparation of lightweight and cheap thermoelectric devices, such as wearable thermoelectric devices.

## 4. Conclusions

In summary, our work demonstrates for the first time the feasibility of fabricating both *p*- and *n*-type SWCNT/PEI composites with enhanced thermoelectrical properties by modulating carrier type via simple mixing techniques. The addition of PEI and Nafion increased the dispersion of the SWCNTs and improved the electrically percolated SWCNT network as a carrier transport pathway. The addition of 13 wt.% PEI modulates the carrier type from *p*- to *n*-type. The Seebeck coefficient of the SWCNT/PEI composite was changed from 49 to −40 μV/K under the experimental range of PEI content. High PFs were obtained with values of 40.5 and 25.5 μW/(m·K^2^) for *p*- and *n*-type SWCNT/PEI composites, respectively. A TE generator consisting of three thermocouples of *p*- and *n*-type SWCNT/PEI composite films delivered an open circuit voltage of 17 mV and a maximum output power of 224 nW at the temperature gradient of 50 K.

## Figures and Tables

**Table 1:** Results of Hall effect test of SWCNT/PEI films at room temperature.

Sample	Carrier Type	Carrier Concentration (n/(cm³))	Carrier Mobility (μ/(cm²/V·s))
SWCNT/PEI-0	*p*	4.6 × 10^20^	5.314
SWCNT/PEI-2	*p*	3.7 × 10^21^	0.252
SWCNT/PEI-15	*n*	9.3 × 10^20^	0.814
SWCNT/PEI-20	*n*	2.6 × 10^22^	0.057

**Table 2:** A summary of some high-performance flexible thermoelectric devices.

TE Units	Device Dimensions	ΔT	Output Voltage	Output Power	Comment	Ref.
*p*-type: poly(3,4-ethylenedioxythiophene):polystyrenesulfonate (PEDOT:PSS)	14 thermocouples	50	12 mV	16.8 µW	Parallel connected single-leg structure; screening printing; paper substrate.	[40]
*p*-type: PEDOT:PSS *n*-type: N-doped graphene	8 cm × 10 cm	10 K	3 mV	≈0.24 mW·m^−2^	Roll-to roll printing.	[41]
*p*-type: carbon nanotubes (CNTs)/poly(3-hexyl thiophene)	41 *p*-type stripes (0.1 cm × 1.5 cm)	10 K	≈32 mV	32.7 nW	Spray-printing; polyimide substrate.	[42]
*p*-type: CNTs/PEDOT:PSS *n*-type: CNTs/PEDOT:PSS treated by hydrazine	14 thermocouples	10 K	8 mV	0.43 µW	Wet-spinning process.	[43]
*p*-type: PEDOT-coated textiles *n*-type: *n*-type CNTs	5 thermocouples	100 K	≈22 mV	62 nW	Vapor phase polymerization; wearable thermoelectric strain sensor.	[44]
*p*-type: Bi_0.5_Sb_1.5_Te_3_ pastes infiltrated paper; *n*-type: Bi_2_Se_0.3_Te_2.7_ pastes infiltrated paper;	10 thermocouples	35 K	8.3 mV	10 nW	Impregnating method; transparent paper-based thermoelectric generator.	[45]
*n*-type: Bi_2_Te_3_/polyvinyl alcohol hybrid composites	10 stripes (0.2 cm × 2.5 cm)	46 K	24 mV	≈9 µW·cm^−2^	Solid-state reaction and ball milling method.	[46]
*p*-type: polypyrrole film *n*-type: copper tape	7 thermocouples	80 K	336 µV	6.4 pW	Flexible scaleable free-standing polypyrrole films; interfacial chemical polymerization.	[47]
*p*-type: PEDOT film	6 stripes (0.7 cm × 3 cm)	51.6 K	≈5.6 mV	157.2 nW	Simple self-assembled micellar soft-template method; vacuum-assisted filtration; ultrafine PEDOT nanowires.	[48]
*p*-type: poly(acrylic acid)/CNTs *n*-type: PEI/CNTs	30 thermocouples	10 K	21 mV	131 nW	A flexible thermoelectric generator prepared by printing the *p*-type and *n*-type CNTs ink on a curved surface.	[49]
*p*-type: PEI/SWCNTs *n*-type: PEI/SWCNTs	6 stripes (0.5 cm × 2 cm)	50 K	8.6 mV	224 nW	Flexible scaleable PEI/SWCNTs films; simple mixing techniques.	Our work
